# The designing of a transparent hybrid machine learning framework for water leak detection: a systematic review

**DOI:** 10.3389/fdata.2026.1761377

**Published:** 2026-04-24

**Authors:** Chinemerem M. Anozie, Tite Tuyikeze, Ibidun C. Obagbuwa, Fezile Matsebula

**Affiliations:** 1 Department of Computer Science & Information Technology, Faculty of Natural and Applied Sciences, Sol Plaatje University, Kimberley, South Africa; 2 Department of Mathematical Sciences and Computing, Faculty of Natural Sciences, Walter Sisulu University, Mthatha, South Africa

**Keywords:** explainable artificial intelligence, hybrid machine learning, machine learning application, sustainable development, water distribution network, water leakage

## Abstract

**Introduction:**

Global water scarcity is increasingly exacerbated by substantial water losses, with approximately 30% of treated water lost annually due to leaks in aging Water Distribution Networks (WDNs). Addressing this challenge requires advanced and reliable leak detection mechanisms. This study investigates the design of a transparent hybrid machine learning framework aimed at improving the accuracy and effectiveness of water leak detection systems.

**Methods:**

A systematic literature review was conducted following PRISMA guidelines. A total of 27 relevant studies were analyzed, focusing on hybrid deep learning approaches that incorporate data fusion, mixed models, and ensemble techniques for leak detection in WDNs.

**Results:**

The findings indicate that hybrid and ensemble learning techniques are becoming more important in the identification of water leaks. Several studies reported exceptional high performance, with some models achieving up to 99% balanced accuracy by leveraging multiple data modalities. These approaches demonstrate strong resilience and adaptability across varying operational conditions.

**Discussion:**

Despite their high performance, the complexity and “black-box” nature of hybrid models limit their practical deployment. The study highlights the importance of integrating Explainable Artificial Intelligence (XAI) techniques to enhance transparency, interpretability, and user trust. The review concludes that future intelligent leak management systems should combine high-performing hybrid models with XAI to develop efficient, interpretable, and trustworthy decision-support systems that support sustainable water resource management.

## 1 Introduction

Water scarcity and water management are vital topics in tackling a foreseen global water crisis that is bound to occur if resources are not utilized and optimized with care to meet the needs of future generations. Water scarcity can be described as “a condition where water demand exceeds available water supply” wherein demand for water by all sectors, including the environment, cannot be satisfied fully due to the impact of water use on supply or quality of water (Alcamo et al., 2000; Kumari et al., 2021; Vörösmarty et al., 2000).

Globally, it was estimated that in 2020, 126 billion cubic meters were lost due to non-revenue water (NRW), and approximately 30% of treated water is lost annually due to leaks (Liemberger and Wyatt, 2019; Wordsworth, 2023) This raises concern regarding the effectiveness of current methods in the prevention of the global water predicament.

Conventional primitive methods like listening sticks, vibration sensors, and hydrophones all deal with background noise effects that affect accuracy and low performance in leak detection (Islam et al., 2022) whereas other current conventional techniques that produce accurate leak detection include ground-penetrating-radar (GPR), leak noise correlators, pig-mounted acoustic sensors, tracer gas injection, and smart ball technology require expensive equipment, are labor-intensive, and costly (El-Zahab and Zayed, 2019; Puust et al., 2010).

Recent trends in water management and leak detection involve the use of emerging technologies in which water management systems are transformed into intelligent and adaptable infrastructure through the integration of these emerging technologies, such as Artificial Intelligence (AI), Internet of Things (IoT), and Machine Learning (ML) algorithms.

IoT devices, such as smart meters and sensors, facilitate the continuous collection of data from pipelines, valves, and pumping systems. The real-time data is then fed directly into the ML models, allowing dynamic decision-making and enabling early warning or alert systems. This combination of new technologies with AI/ML leads to predictive maintenance, minimizing water loss, reducing repair costs, and extending the useful life of water infrastructures (Bakhtiari et al., 2025). Moreover, these technologies support data-driven water conservation strategies by providing actionable insights into consumption patterns, leak localization, and pressure management.

Data-driven approaches, like AI/ML models, rely on a collection of data to perform statistical analysis for leak detection and do not require any specific in-depth knowledge about a system, and are only dependent on historical data coupled with statistical or pattern recognition tools (Chan et al., 2018; Romano et al., 2014) whilst uncovering relationships between system state variables without using explicit instructions (Wordsworth, 2023). The data-driven strategies used in ML are divided into five categories, namely prediction, classification, clustering, model-based, and statistical methods. Research in the field of water leak detection has used a variety of ML models mainly for classification purposes, where SVM is one of the most used models, followed by Neural Networks and Random Forest (Islam et al., 2022). Real-world problems are complicated, fragile, and may require intelligent and sophisticated models or algorithms to effectively and precisely tackle solutions. Because of the practical importance of these real-world problems, single machine learning models are not sufficient and robust enough to properly and extensively bring solutions to the problems identified. (Wordsworth 2023) pronounce that models do not contain the full complexity of the physical phenomenon, but provide a less complex abstraction that approximates the real system.

The majority of single algorithms or pure ML models are capable of solving the problems they face, but none are completely perfect alone. This can be argued in support of the *No Free Lunch Theorem*, which states that no one algorithm performs optimally across all problem domains; hybrid techniques take advantage of the complementary capabilities of many models. Through the integration of different methods, hybrid methods can take advantage of the strengths of each algorithm and mitigate their limitations (Goldberg, 1989; Wolpert and Macready, 1996). A hybrid algorithm that combines optimization and machine learning techniques is an effective strategy that uses the advantages of both methodologies to provide a powerful framework to tackle complex problems, which leverages optimization capabilities to guide the learning process and improve the accuracy and efficiency of decision-making. By merging balancing algorithms, it is possible to exploit their strengths and overcome limitations, which leads to improved overall performance (Azevedo et al., 2024). However, since hybrid models and algorithms tend to be complex, there is an essential need for explainability and interpretability, especially in the field of water leak detection and management. The future and success of these mixed algorithms lie in their integration with deep learning, transparency as a means of increased explainability, and real-time adaptation.

Therefore, the objective of the systematic review is to search for the best means to design a transparent hybrid machine learning framework that integrates multiple algorithms for leak detection and early leak detection. The research is organized into the following sections, where Section 2 defines the scope and methodology of carrying out the systematic review, and Section 3 thematically discusses and compares the literature found within the search criteria. Section 4 summarizes findings and results drawn. Section 5 describes future directions, and Section 6 concludes the paper.

## 2 Methodology

A structured methodology was followed to systematically analyze the use of hybrid machine learning techniques and mixed methods to detect and locate leaks in a Water Distribution Network (WDN). This approach adheres to the established guidelines for systematic reviews in computer science, artificial intelligence, and machine learning (Saeidmehr et al., 2024). The methodology includes defining a review protocol, conducting a comprehensive search, screening results for eligibility, analyzing the quality of studies, and data extraction and synthesis.

### 2.1 Systematic review protocol

A review protocol was formed to establish a smooth process for identifying, selecting, and synthesizing studies. This was done through the use of PRISMA (Preferred Reporting Items for Systematic Reviews and Meta-Analyses) guidelines that outline the eligibility criteria, search strategy used, and selection process to ensure transparency and validation of the review (PRISMA, 2020).

The primary research question guiding this review is: *How can a transparent hybrid machine learning framework enhance the accuracy and efficacy of detecting and localizing leaks and early leaks in a Water Distributed System as compared to single machine learning models and conventional methods?*

### 2.2 Comprehensive search

To ensure a comprehensive literature review, a structured search strategy was implemented. The search was first conducted by brainstorming relevant keywords such as “water leak detection,” “hybrid machine learning,” “explainable AI,” and “Water Distribution Network” with alternative synonyms such as “water loss,” “artificial intelligence,” and “Water Distribution System,” whilst comparing keywords to relevant studies.

The type of search strategy then utilized in the comprehensive search was a single line strategy, which included Boolean terms such as “OR,” “AND,” with the associative key terms. The following key terms with the mentioned search strategy were inserted into the databases listed below (“water leakage” OR “water loss” OR “water burst”) AND (“water distribution network” OR “water distribution system” OR “water supply system”) AND (“hybrid machine learning” OR “explainable artificial intelligence” OR “hybrid methods” OR “data-driven approach”).

Once the keywords for the systematic review were identified through a review protocol, the search was then conducted in suitable and available databases that yielded results with quality search feedback through a title and abstract selection of the search results. Table 1 summarizes the initial selected number of records from each database, with its number of search results.

**Table 1:** Summary of databases and initial search results identified.

Database	No of search results	Selected no of records	Search fields
ScienceDirect	12	9	All content, water research journal
Scopus	35	16	All content, limited to the computer science area of subject
Taylor & Francis	11	3	Anywhere
IEEE Xplore	144	11	All metadata
EBSCO Host	8	2	Abstract, title, and index terms

### 2.3 Inclusion and exclusion criteria

A set of criteria was established to focus on relevant and valuable research to form a good analysis for the paper. Studies were evaluated based on the following factors and are tabulated in Table 2.

**Table 2:** Summary of inclusion and exclusion criteria.

Criteria	Inclusion	Exclusion
Publication year	2010–2025 (past 15 years)	Before 2010
Source type	Journals, conference papers, peer reviews, and research articles	Reports
Empirical evidence	- Performance metrics (accuracy, test-score, etc.) - Studies that demonstrated valid results in the detection and location of early/small leaks in WDNs or WDSs	- Studies that have not reported relevant outcome measures - Studies that did not prove effective or had a tangible impact on the field of water leakage and detection
Focus on the type of hybrid or mixed approaches in water leak detection	- Studies that utilize hybrid or mixed methods and approaches to detect water leakage and/or predict location - Studies on XAI with hybrid water leak detection	- Studies that focused only on single machine learning models or leak methods and approaches as performance measures - Studies that focused more on hardware or the IoT aspect of a Smart Water Management (SWM) System

#### 2.3.1 Inclusion criteria

° Studies published between 2010 and 2025.
° Journals, conference papers, reviews, and research articles.
° Research that validated the increased efficacy of detecting and locating leaks through established performance metrics.
° Research also demonstrated valid results in the detection and/or location of early leaks in WDNs or WDSs.

#### 2.3.2 Exclusion criteria

° Studies that have not been proven effective or have not had a tangible impact on the specific time criteria.
° Studies that have not reported relevant outcome measures.
° Studies that focused only on single machine learning models or single leak detection methods and approaches as a performance outcome.
° Studies that focus more on the ‘integration of the ”smart' or IoT aspect of a water leak detection system.

### 2.4 Selection process, screening, and quality assessment

**Figure 1:**
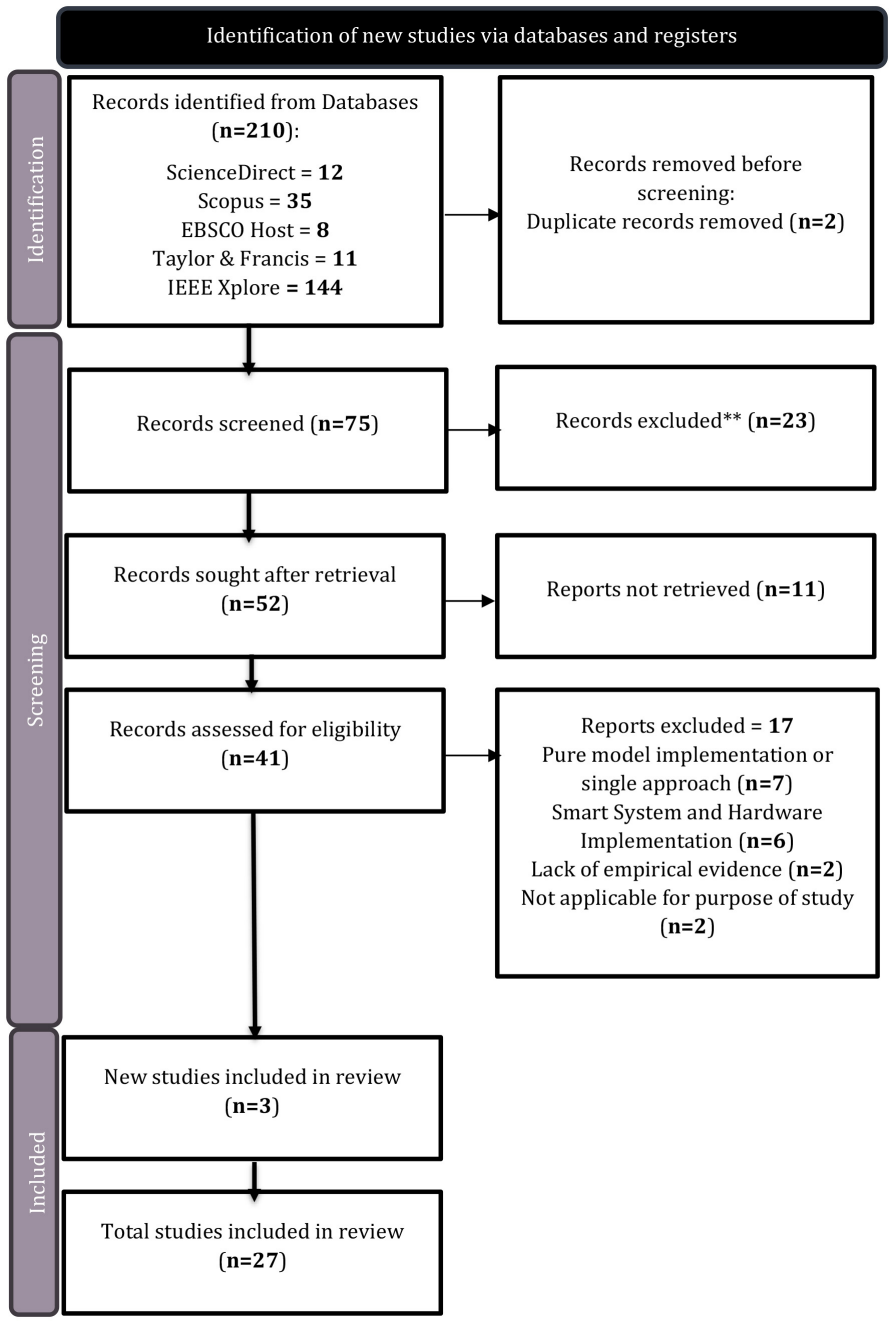
Prisma flowchart.

The total initial search yielded 210 results and was filtered using a title and abstract screening process to assess the relevance of the sources, which then reduced it to 41 results. The screening process included reading the articles in full and evaluating each source material against the inclusion and exclusion criteria as listed in Table 2. Once a review paper met the inclusion criteria, it was subjected to the Critical Appraisal Skills Programme (CASP) quality assessment checklist (CASP Checklists, 2025), ensuring that bias was not identified in studies and that the results of the studies and reviews were valid, further reducing the pool to 24 studies. Three additional and individual studies were reviewed from references found in the reviewed studies that went under evaluation and were added to the final 24 studies, resulting in a total of 27 included in the literature review of this paper. Figure 1 depicts the complete selection and screening process using the PRISMA flowchart. The selection process ensures a good overview of the types of hybrid and mixed techniques used in the literature, with the aim of identifying a design of a hybrid transparent framework for leak detection and possible localization in a WDN.

### 2.5 Data extraction and synthesis

**Figure 2:**
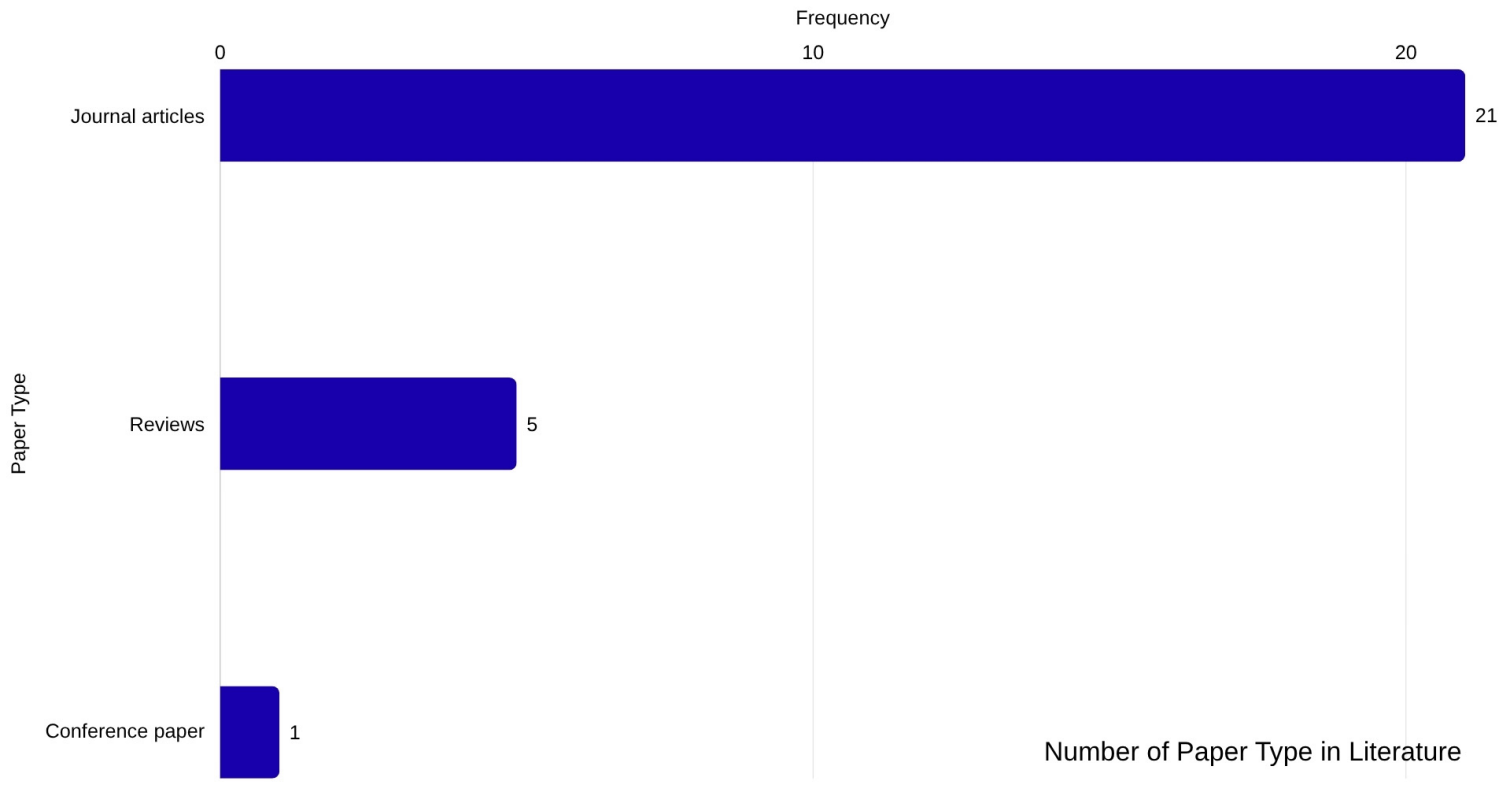
Number of study types included in the literature.

The number of studies included in the review consists of a 3.7% conference paper, 18.5% review papers, and 77.7% journal articles for this research. The number of each study type can be depicted in Figure 2. The data extraction method focused on identifying the type of mixed or hybrid method utilized among studies, the type of dataset and features used for validation, as well as identifying the studies contribution and limitations in the area of water leakage detection. The purpose of the extraction was implemented to arrange findings thematically and to allow for clear comparisons of techniques and their respective advantages and disadvantages when designing a hybrid machine learning model for leak detection for water utilities.

The data was then synthesized into a thematic review of the literature to understand current knowledge, trends in water leak detection and management, and to identify gaps and controversies in the research field.

### 2.6 Analysis and synthesis approach

A thematic synthesis allowed for the identification of patterns, themes, and research gaps among the studies included in the review through a comparative analysis that was carried out to determine the following:

° The type of hybrid/mixed model combinations and approaches
° The type of data acquisition and feature extraction used
° The use of Explainable AI (XAI) in hybrid water leak detection
° The performance and effectiveness of the best models overall
° The knowledge gaps and controversies identified amongst literature.

## 3 Thematic review of literature

### 3.1 Hybrid/mixed model combinations and approaches

A bibliometric analysis of the papers shows a substantial concentration of research focused on data-driven and hybrid ML methodologies for water leak detection and management, with a marked acceleration output of articles published during the last 5 years. The publications were relatively low between 2017 and 2020, accounting for 6/27 articles and significantly high in the years 2021 and 2025, with 2023 papers showing more frequently in the review. Earlier foundation of the research shows a pattern in mixed model/data-driven methods and mathematical approaches. A persistent challenge was then identified in 2020 papers, which was the scarcity of high-quality labeled datasets that promoted research into synthetic data generation and purely data-driven approaches. The most significant trend was a surge in publications detailing advanced hybrid ML and fusion techniques, with the majority of the relevant papers appearing between 2021 and 2025. The clusters of these involve the implementation of hybrid ML techniques for leak characteristics through the means of deep learning and ensemble methods. This suggests that methodologies concerning ensemble or hybrid deep learning leak detection are the subject of very recent and ongoing scholarly emphasis in the complex and unpredictable environments of WDNs.

#### 3.1.1 Conceptual background: the evolution from data-driven and hybrid models

Historically, leak detection relied on model-based methods that needed a well-calibrated hydraulic model of a network (Rajabi et al., 2023; Romero-Ben et al., 2023; Soldevila et al., 2017), however, creating and maintaining those models can be time-consuming, challenging, and often impractical for several water companies. (Chan et al. 2018) indicate that the challenge in the model-based approach is the uncertainty of model parameters, such as a pipe object, where the leak detection method often assumes that the condition of the pipe remains the same over time. For this reason, a shift has driven toward data-driven approaches that learn directly from historical sensor data without needing knowledge of a hydraulic system (Martinez-Rios et al., 2024; Rajabi et al., 2023; Romero-Ben et al., 2023). Hybrid or mixed model-based and data-driven methods have emerged as a powerful paradigm. Among these mixed model-based and data-driven approaches, a hydraulic model is used offline to generate simulated leak scenarios, which can be used as labeled training data for ML classifiers that support physical understanding from a hydraulic model while benefiting from the pattern-recognition capabilities of machine learning (Rajabi et al., 2023; Romero-Ben et al., 2023).

#### 3.1.2 Hybrid and ensemble model architectures

Modern leak detection models use ensemble learning (MEL), in which multiple models are constructed to solve the same problem, which improves the accuracy and robustness of the model, as well as hybrid deep learning methods that integrate the use of AI. Some models witnessed in the literature tend to have a mix of both MEL and hybrid deep learning methods (Martinez-Rios et al., 2024; Zhou et al., 2021; Zese et al., 2021) while some studies found, utilized mixed model-based or mixed data-driven methods that can be elaborated upon in Table 3.

**Table 3:** Summary of mixed model-based/data-driven methods identified in the literature.

References	Mixed model-based/data-driven method	Performance metrics	Functionality	Brief description	Limitations
(Dogani et al. 2020)	A hybrid meta-heuristic algorithm combining the Red Deer Algorithm (RDA) and Simulated Annealing (SA).	The proposed hybrid algorithm was shown to be superior to the standard NSGA-II algorithm across several assessment metrics.	Bi-Objective Stochastic Optimization of the urban water supply system.	The study presents a mathematical model that balances the minimization of remodeling costs with the minimization of water leakage, using the hybrid RDA-SA algorithm to solve the optimization problem under uncertainty.	The model omits certain technically relevant hydraulic details, such as pressure heads and the roughness of pipes.
(Menapace et al. 2020)	A two-phase Stochastic-Hydraulic Time Series Generator (SHtsG) methodology using a Distributed Fully Pressure-Driven Model (DFPDM).	Performance metrics are not provided as this is a data generation methodology, not a detection model. The main contribution is the realistic modeling of water requests and bursts.	Synthetic dataset generation for burst detection models.	This study generates realistic water consumption time series data for training detection models. It first stochastically models water requests by superimposing patterns and randomness, then uses a hydraulic simulation (DFPDM) to model demand, background losses, and bursts.	The primary limitation is that the study developed a data generation methodology but did not extend it to the direct application of testing or developing a specific anomaly detection algorithm with the generated data.
Muniz Do Nascimento and Gomes-Jr. (2023)	Semi-supervised learning for outlier detection combined with Automated Machine Learning (AutoML), using Self-Organizing Maps (SOM) and Local Outlier Factor (LOF).	Achieved an *F*-score mean of 0.36 for SOM and 0.35 for LOF, outperforming standard methods.	Low-Cost automatic water leakage detection.	The study adapts unsupervised algorithms (SOM, LOF) to a semi-supervised scheme using maintenance logs as proxies for anomalous data. AutoML is used for automatic hyperparameter optimization to create an effective, low-cost model.	The dataset was unreliable as maintenance logs are imprecise. The modeling lacked comprehensive feature engineering, and the solution has not been implemented in a live production environment.
Quinõnes-Grueiro et al. (2018)	Unsupervised framework using Principal Component Analysis (PCA) on pre-processed pressure signals.	Achieved an overall performance of 85.28% Fault Classification Rate (FCR), which was comparable to supervised methods.	Leak detection and location.	This unsupervised framework relies only on pressure-sensor data from normal network operations. It divides the network into zones, preprocesses pressure data to create stationary signals, and then applies PCA to build a statistical model of normal behavior for detecting and localizing leaks.	Limitations include a dependence on simulated data, reduced reliability for leaks in high-demand nodes, and the need for more extensive sensor coverage to improve robustness.
(Soldevila et al. 2017)	A mixed model-based and data-driven methodology using a Bayesian classifier.	The Bayesian classification method achieved a 91.59% accuracy score, outperforming k-NN and angle methods.	Leak location	The approach uses a hydraulic model (EPANET) in an offline phase to generate data and calibrate the model. In the online phase, real-time pressure residuals are fed into the Bayesian classifier to determine the leak's location.	The method has practical difficulty in resolving leak locations when different nodes produce similar pressure residuals, leading to “overlapped nodes” that cannot be distinguished from one another.
(Ulusoy et al. 2022)	Mixed data-driven method combining Evolutionary Algorithms (EA) and gradient-based mathematical optimization.	Achieved a low Average Zone Pressure (AZP) of 29.9 m.	Pressure management and leakage minimization.	Investigates an adaptive District Metered Area (DMA) aggregation strategy to jointly minimize pressure-induced leakage and maximize resilience by optimizing the control settings of Pressure Reducing Valves (PRVs).	The optimization methods may not scale well for large Water Distribution Networks (WDNs) with many discrete decision variables.
(Xing and Sela 2019)	Time Series Data Mining (TSDM) approach using modified CUSUM, Dynamic Time Warping (DTW), and k-means clustering.	The study revealed consistent pressure transient patterns and provided a fast, efficient way to analyze high-frequency signals.	Unsteady pressure pattern discovery.	A two-step TSDM approach is used to process high-frequency pressure data. First, a modified CUSUM algorithm detects pressure changes. Then, the extracted pressure transients are clustered using DTW and k-means to discover representative patterns.	There is a potential challenge in analyzing large volumes of data, and further research is needed to correlate the discovered pressure transients with specific pipe failures like leaks and bursts.

##### 3.1.2.1 MEL methods

Stacking, bagging, and boosting are common ensemble algorithms typically used in MEL architecture. (Ravichandran et al. 2021) used an ensemble of Gradient Boosting Tree (GBT) classifiers, aggregated using a bagging algorithm, which outperformed baseline classifiers like KNN and ANN and demonstrated significant performance and robustness over conventional ML and rule-based methods. It has been shown to significantly reduce false positives as compared to conventional methods, which is critical in the area of water leak detection because it reduces the cost of investigating false alerts in water utilities. Whereas, with (Zhou et al. 2021), proposed a novel ensemble transfer learning technique of a one-dimensional convolutional neural network that deviates from typical MEL approaches by integrating base learners (TL1DCNNs) where their weights are mathematically optimized using Particle Swarm Optimization (PSO) through minimizing the sum of similarity between base learners to reduce generalization. (Saravanabalaji et al. 2023) experiments with several conventional ML models, namely Logistic Regression (LR), XGBoost, SVM (linear), SVM(RBF), Random Forest (RF), AdaBoost, and Neural Network (NN), to find the best combinations of hybrid ML models amongst them. This was carried out by taking the weak learners' predictions collected by an arithmetic mean, with the individual models weighing their performance appropriately. The method performed by the researchers capitalizes on the diverse strengths of each base model. Table 4 lists a summary of all MEL methods identified in the literature.

**Table 4:** Summary of MEL methods identified in the literature.

References	MEL method	Performance metrics	Functionality	Brief description	Limitations
(Lucin et al. 2021)	Random Forest Classifier (an ensemble method that uses the bagging of decision trees).	The accuracy surpassed 90% when considering the top few predictions.	Leak localization	The study used a Random Forest model trained in simulated EPANET leak scenarios to predict leak locations. A key contribution is a novel pipe segmentation approach that allows the model to localize leaks occurring between network nodes, not just at the nodes themselves.	Increasing demand variation rapidly decreases the exact prediction accuracy of all models when implemented in a real-world environment.
(Martinez-Rios et al. 2024)	A Late Fusion (LF) ensemble of SVMs, which uses a majority voting mechanism to combine predictions from unimodal models trained on accelerometer, pressure, and acoustic data.	The LF model achieved 93.33% accuracy for looped networks and 95.83% for branched networks.	Leak detection and classification	This approach uses the Wavelet Scattering Transform (WST) for feature extraction as an alternative to deep learning. The LF model combines predictions from multiple SVMs to perform multi-class classification, identifying the *type* of leak (e.g., orifice, crack) from laboratory data.	The performance of the hydrophone data was negatively affected due to background noise interference collected from a laboratory testbed dataset.
(Ravichandran et al. 2021)	A Multi-strategy Ensemble Learning (MEL) approach that combines multiple Gradient Boosting Tree (GBT) classifiers using a parallel bagging algorithm.	The ensemble model reduced the False Positive Rate to 0.16% and achieved an overall accuracy of 99.84% with 100% sensitivity.	Leak detection	This work presented an acoustic leak detection system using an ensemble classifier trained on more than 14,000 samples from water mains in the USA and Canada. A key challenge was the highly imbalanced dataset, which was addressed using semi-supervised training with maintenance logs as proxies for leak events.	High asymmetrical data composition due to the absence of real leak events in the dataset as these abnormalities were rather simulated and combined with real water data.
(Saravanabalaji et al. 2023)	A weighted hybrid ensemble model (Hybrid Model-4) that combines six machine learning models: Logistic Regression, SVM, AdaBoost, Random Forest, Neural Network, and XGBoost.	The hybrid model achieved a testing accuracy of 99.3%, outperforming conventional ML techniques.	Leak detection	This study developed a system for detecting water leakage using acoustic signals collected in a laboratory setting. The methodology involved processing signals with the Fast Fourier Transform (FFT) and reducing dimensionality with Principal Component Analysis (PCA) before classification.	The results were achieved using a laboratory scale-up model due to resource constraints.
(Zese et al. 2021)	An ensemble of 1D CNNs, where the final model's weights are averaged using the Polyak-Ruppert approach after a 10-fold cross-validation training process.	Achieved accuracy, precision, and recall ranging from 92% to 96%.	Leak detection and identification of small leaks	This study focuses on detecting and classifying the magnitude of intra-domestic water leaks (e.g., small, medium, large) using time-series data from smart meters in Italy. The model demonstrated the ability to detect very small leaks ( ≤ 1 L/h) that are often missed by other methods.	Difficulty distinguishing between the absence of leaks and small leaks (< =1 L/h) due to small leaks that exhibit characteristics of a “sawtooth behavior” in a 5-min time series.
(Zhou et al. 2021)	An Ensemble Transfer Learning One-Dimension Convolutional Neural Network (Ensemble TL1DCNN), where the weights of the base learners are optimized using Particle Swarm Optimization (PSO).	Achieved 90.5% precision, 91.8% recall, and a 90.4% *F*-score.	Leak detection and localization	This study proposes an ensemble of 1D-CNNs that leverage transfer learning to improve performance, even with smaller datasets. The approach was validated using a dataset generated via simulation software based on negative pressure wave signals.	A simulated dataset generated by the pipeline simulation program Flowmaster V7 was used to assess the ensemble Transfer Learning One-Dimensional Convolutional Neural Network (TL1DCNN) technique, which produces noiseless base data on a simplified WDN model.

##### 3.1.2.2 Hybrid deep learning methods

Fusing deep learning with traditional ML has yielded high-performing models. For example, (McMillan et al. 2024) proposed a hybrid framework that combines a domain-informed variational autoencoder (VAE) for dimensionality reduction with an SVM for classification of a water flow series data into leakage and non-leakage categories, which achieved an accuracy score of 98% in distinguishing the different event scenarios. Other innovative hybrid deep learning methods include the use of an LSTM-GAN model, where the Short Time Fourier Transform (STFT) technique is utilized to draw a spectrogram from a collected data sample. The LSTM-GAN model is then trained on the acoustic signals to generate the leak dataset and enhance its leak detection capabilities (Liu et al., 2024). Another example of a hybrid generative adversarial network (GAN) can be seen in a study carried out by (Rajabi et al. 2023), which utilizes CDCGAN for image-based leak detection and localization (LD & L) in a WDN. A good enough score of approximately 70% accuracy was achieved in real-time leak detection and was regarded as robust against environmental uncertainty. (Tornyeviadzi et al. 2023) utilize semi-supervised learning by training a BiLSTM deep AE solely based on leak-free scenarios, and it has demonstrated significant superiority over traditional methods once validated on residential, commercial, and industrial District Metering Areas (DMAs). It was also proven faster in detecting unreported leakage within a space of 1–4 days, as compared to traditional methods that could usually take up to 15 days. More complex systems have been developed for LD&L using deep hybrid ML methods like an ensemble 1D CNN architecture using a Polyak Ruppert approach when it comes to detecting and classifying different magnitudes of leaks (Zese et al., 2021). Table 5 summarizes all hybrid deep learning models evaluated for the literature review.

**Table 5:** Summary of hybrid deep learning models identified in literature.

References	Hybrid deep learning model	Performance metrics	Functionality	Brief description	Limitations
(Atanane et al. 2023)	Transfer Learning on five Convolutional Neural Network (CNN) variants, with EfficientNet showing the best performance.	Up to 97.45% testing accuracy and a 97.63% F1 score.	Leak detection	This study proposes a resource-efficient solution for real-time leak detection using TinyML. The methodology involves transforming acoustic data into scalogram images and applying transfer learning to CNN models optimized for deployment, through a process called quantization so that it can be implemented on low-cost devices like the Arduino Nano 33 BLE.	A noticeable gap in the integration of TinyML methodologies and the actual implementation of the models in a real-world environment had not been highlighted.
(Leonzio et al. 2025)	A complex-valued Convolutional Neural Network (CNN) with a multi-sensor, feature-level fusion strategy (hydrophone and accelerometer), followed by a complex-valued Multi-Layer Perceptron (MLP) for classification.	Achieved a balanced accuracy of 99% with sensor fusion, showing significant improvement over three baseline models in both controlled and real-world scenarios.	Leak detection and classification	The four-step pipeline involves converting raw sensor signals into the frequency domain (STFT), extracting complex features with the CNN, fusing features from both sensors, and classifying the output as “leak” or “no leak” with the MLP. The method was validated on both a laboratory dataset and a real-world dataset from the Hong Kong WDN.	The multi-sensor fusion strategy was only fully demonstrated on the laboratory dataset due to a lack of perfectly synchronized sensor data in the real-world dataset. Real-world performance may also be affected by environmental noise and flow fluctuations.
(Liu et al. 2024)	A hybrid Long Short-Term Memory generational adversary Network (LSTM-GAN).	The enhanced model achieved 94.02% testing accuracy.	Leak detection	The study addresses data scarcity by using an LSTM-GAN to generate synthetic acoustic leak signals, thus enriching and diversifying the training dataset. The model was trained and validated using acoustic signals collected from real-world Water Distribution Networks (WDNs) in Hong Kong.	Limited diversity in generated data due to a lack of consideration for no-leak signals and the potential introduction of outliers with increased generated samples.
(Ma 2024)	Transductive Long Short-Term Memory (T-LSTM)	Achieved 98.3% accuracy, 98.2% precision, 98.2% recall, and 98.1% F1-score.	Leakage detection	The T-LSTM model processes water pressure data from residential WDN. Its key feature is a transductive method that creates new data sequences by analyzing neighboring data points, making the model robust to sudden input data changes and allowing it to detect both small and large leaks.	The paper notes that future work will focus on enhancing the detection process by incorporating more advanced deep learning techniques.
(McMillan et al. 2024)	A hybrid framework combining a domain-informed Variational Autoencoder (VAE) for dimensionality	The framework achieved 98.2% classification accuracy.	Leak detection	This approach rapidly identifies leaks at the District Meter Area (DMA) level by reducing high-dimensional water flow time-series data into a	The data-driven approach used in the study relies on the quality of the data used in training due to its lack of actual practicality
	reduction and a Support Vector Machine (SVM) for classification.			two-dimensional latent space using a VAE. An SVM then classifies the data as leakage or non-leakage. The model was trained on data from more than 2,500 DMAs in the UK.	and implementation of the framework in a real-world environment.
(Naith and Mancy 2025)	An IoT-Enabled Hybrid Multi-Agent Deep Reinforcement Learning (MADRL) framework combined with SHapley Additive exPlanations (SHAP) for eXplainable AI (XAI).	Achieved 30% reduction in water loss, 88% pressure stability on the LeakDB benchmark and an 87% SHAP consistency score.	Leak detection and management	This framework integrates IoT data, a Multi-Agent Deep Reinforcement Learning (MADRL) control model, and an XAI component to create a transparent system for urban water management. Rather than simple detection, it focuses on real-time control and leak mitigation, providing human-understandable explanations for its actions.	Framework relies heavily on benchmark and simulated data, necessitating future deployment in real-time settings to test performance under conditions like sensor faults and delayed feedback, whereas computing SHAP values is expensive in high-dimensional or multi-agent environments, because it poses scalability issues for networks exceeding 1,000 nodes.
(Rajabi et al. 2023)	Conditional Deep Convolutional Generative Adversarial Networks (CDCGAN), based on the pix2pix architecture.	Approximately 70% accuracy in real-time leak detection.	Leak detection and localization	This method converts hydraulic pressure data into images and uses a CDCGAN for image-to-image translation to spot anomalies. The Structural Similarity Index (SSIM) is used to detect leaks (globally) and localize them (locally). The approach was validated on the L-Town benchmark problem.	The performance of the model can be affected by the complexity and size of the water distribution network.
(Tornyeviadzi et al. 2023)	A Bidirectional Long Short-Term Memory (BiLSTM) deep Autoencoder (AE).	Achieved a high precision score of 0.8621 and detected leaks in 1–4 days, compared to 15 days for traditional methods.	Leak detection	This study uses a semi-supervised learning approach, training the BiLSTM deep autoencoder solely on normal night-flow data (2–4 a.m.) from a real-world WDN in Norway. Leaks are identified as anomalies when the reconstruction error exceeds a set threshold.	The limit highlighted was the stochastic demand; for instance, using an appliance late at night complicates the stability of the timeframe in DMAs.
(Xu et al. 2025)	A “Hyperclustering” framework that integrates deep (CNN) and shallow learning techniques using hypergraph learning, combined with a Gaussian Mixture Model (GMM) for probabilistic ranking.	Outperformed 16 other models, achieving an Average Precision (AP) of 33.28% on the DAC-TSD dataset, 27.57% on the MOHR dataset, and 26.47% on their compiled dataset.	Leakage detection	The framework fuses deep features (e.g., from CNN images) and shallow features (e.g., environmental data) using a hypergraph to model complex relationships. A GMM then ranks potential leakage areas to improve accuracy and identify	The current implementation may not be fully optimized for live or real-time feedback loops.
				high-risk zones. The method was validated on datasets of subway tunnel images captured by a Mobile Laser Scanning (MSL) system.	

### 3.2 Data acquisition and feature extraction

The foundation of any data-driven leak detection system is the quality and nature of the input data and the methods used to extract meaningful features from them. Research demonstrated that the choice of data influences a model's ability to detect different types of leaks (Qi et al., 2024; Martinez-Rios et al., 2024).

#### 3.2.1 Sensor data modalities in WDNs

##### 3.2.1.1 Flow and pressure data

As the most commonly used data in WDNs, flow and pressure measurements form the basis of many leak detection systems. (Rajan and Li 2025) argue that flow sensors prove to be more effective for leak detection, whereas pressure-based sensors can be limited to transmission distance, signal strength, and sensor cost; hence, flow-based sensor data and methods may give practical and cost-effective solutions for automated leak management. This type of data is considered useful for detecting large bursts or significant changes in hydraulic systems (Menapace et al., 2020; Rajabi et al., 2023). However, having unsteady pressure and flow can be influenced by numerous factors other than leaks that include as demand variability and operational changes (Xing and Sela, 2019), making it difficult to distinguish small leak signals from normal system noise. For this reason, many advanced systems use flow and pressure data in conjunction with more sensitive sensor types or as part of hybrid models for leak detection (Martinez-Rios et al., 2024; Rajan and Li, 2025).

##### 3.2.1.2 Acoustic and vibration data

For these data modalities, it is considered highly effective for leak detection because leaks generate distinct acoustic emissions (AE) and vibration signals that propagate through pipe material, which can be captured using accelerometers (Saravanabalaji et al., 2023). In support of this, (Martinez-Rios et al. 2024) also demonstrated that accelerometer data, when properly processed, can yield very high classification accuracy, as shown in their study of an SVM trained on feature extraction from using one of four sensors including accelerometers (that collect vibrational signals) which achieved accuracies of 89.58% in a looped network and 96.6% in a branched network, outperforming models based on dynamic pressure and hydrophone data. Hydrophones, similar to acoustic sensors, capture acoustic signals within water distribution systems (WDSs), although their performance can be heavily impacted by background noise. Due to hydrophones' high sensitivity, they are particularly suitable for identifying early or very small leaks, this can be read in the study carried out by (Qi et al. 2024) where it successfully detected leaks as small as 0.03 L/s.

**Figure 3:**
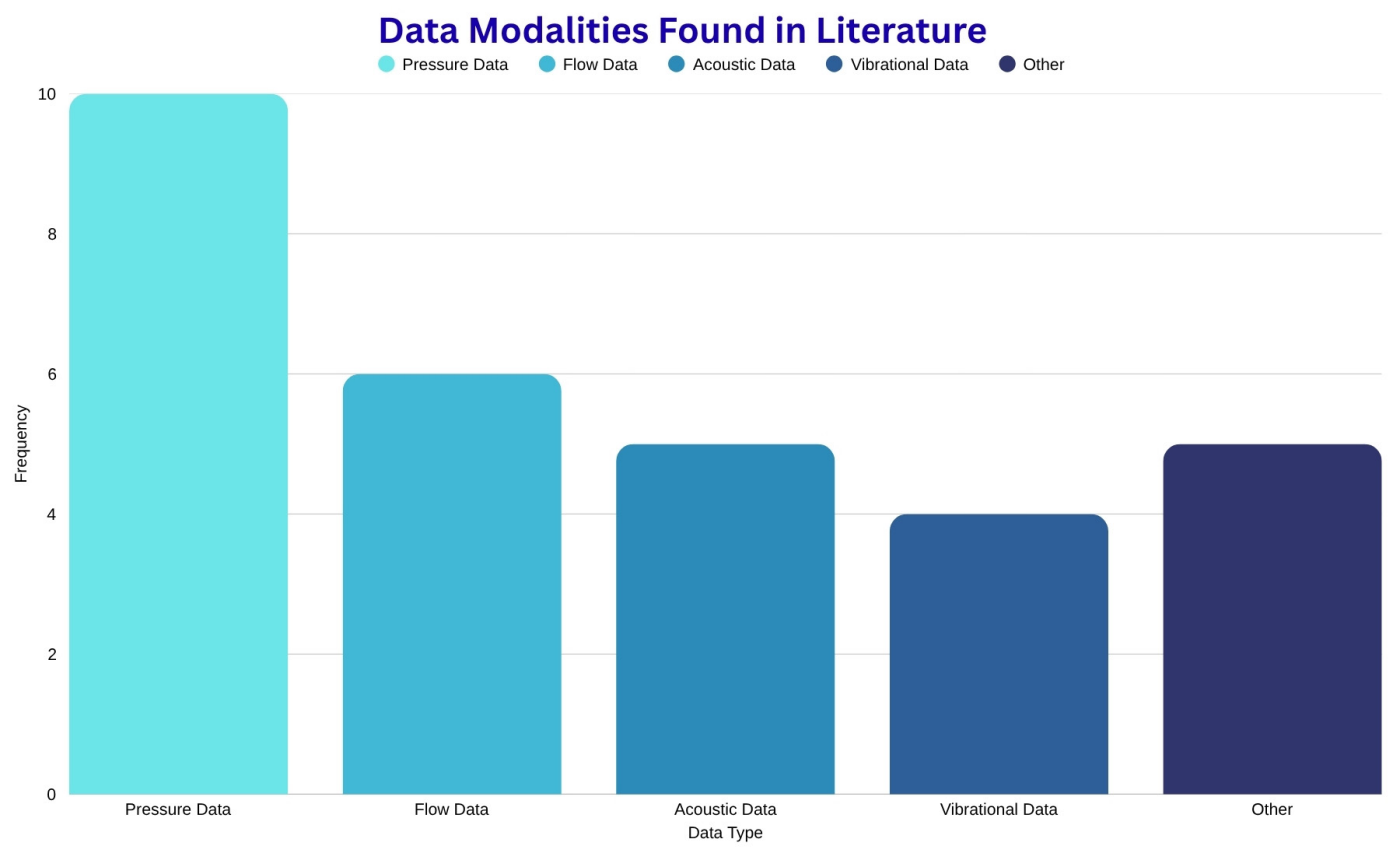
Number of data modalities used in the literature.

**Figure 4:**
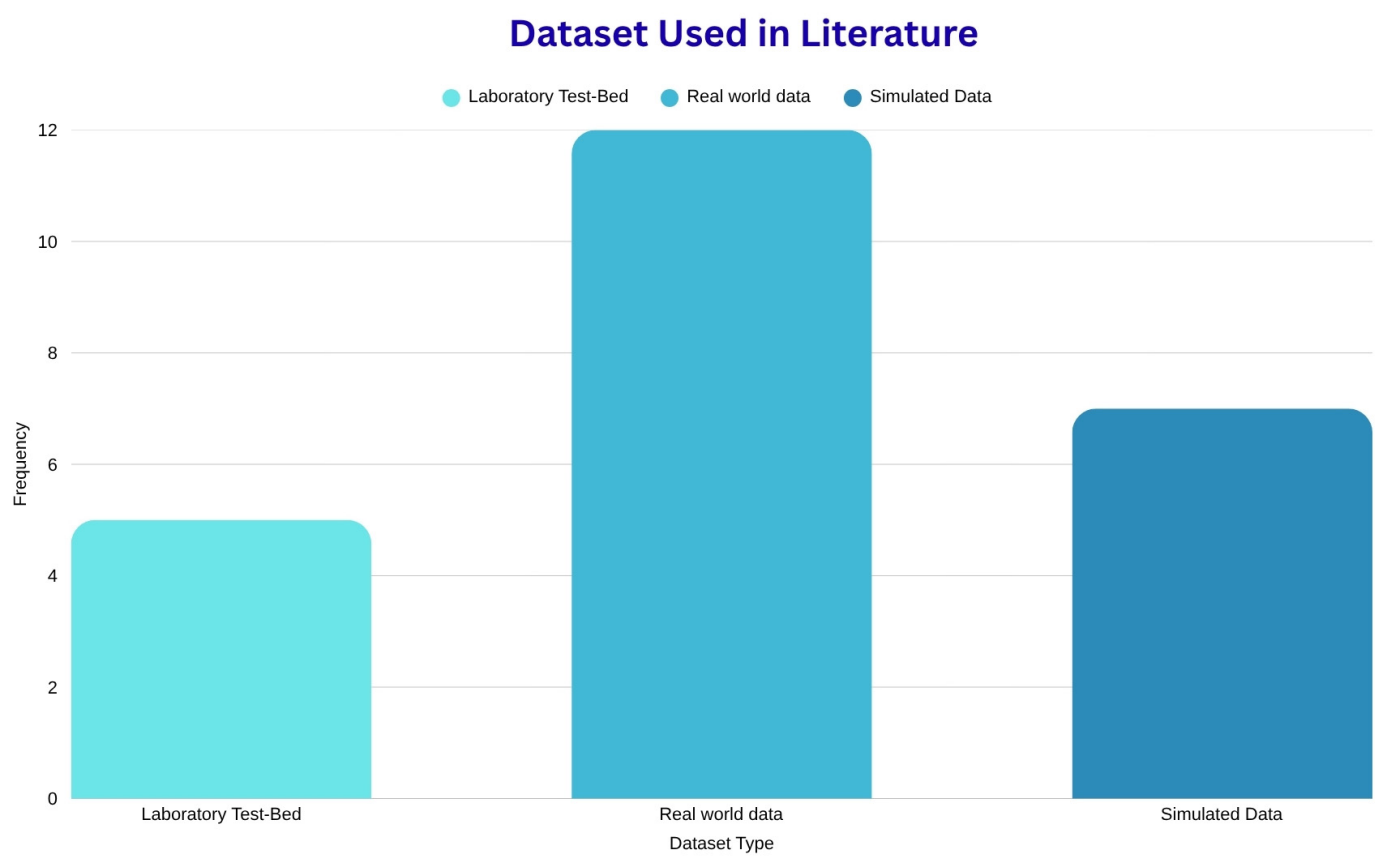
Number of dataset types used in the literature.

A count summary of the different data modalities found in the literature is visualized in Figure 3 as well as the count of the type of dataset used in each literature drawn in Figure 4.

In Figure 3, a noticeable trend toward pressure data used in hybrid ML models for water leak detection can be detected, followed by flow, acoustic, and other data. “Other” in the illustration, as identified, is coupled with data images (Xu et al., 2025), video frames, water demands, and system parameters (Dogani et al., 2020), etc., whereas the lowest type of data modality used is vibrational data.

Although the use of real-world data seems to be large and may seem to undermine the issue of dataset scarcity of water leak anomalies (Menapace et al., 2020), as shown in Figure 4, it is important to note that combining both simulated and laboratory data results in the same number of real-world datasets. It is also important to keep in mind that most of these real-world datasets had minimal leak scenarios captured in them. Table 6 gives an overview of the commonly used benchmark and state-of-the-art datasets, as well as their limitations in the literature.

**Table 6:** Description of commonly used benchmark datasets.

Benchmark/state-of-the-art dataset	Primary use/type	Authors who utilized the dataset in the literature	Key limitation
Aghashahi et al. (2022)	Laboratory test bed (acoustic, pressure & vibration)	(Leonzio et al., 2025; Martinez-Rios et al., 2024)	Laboratory conditions might not accurately capture the full complexity of a real-world WDN
LeakDB benchmark	Simulated (hydraulic sensor reading)	(Naith and Mancy, 2025)	Depending solely on simulations leaves a gap in capturing the noise and complexity of real-world conditions
Hong Kong WDN dataset	Real world (acoustic and vibration)	(Leonzio et al., 2025; Liu et al., 2024)	Possible degradation in performance resulting from remaining synchronization inaccuracies or challenging ambient noise conditions
Hanoi WDN	Simulation (pressure data)	(Lucin et al., 2021; Quinõnes-Grueiro et al., 2018; Soldevila et al., 2017)	Models trained exclusively on node leaks have difficulty forecasting leaks that arise within a pipe segment
L-TOWN benchmark	Simulation/real-world comparison (pressure data)	(Rajabi et al., 2023)	Methods are limited by the requirement for accurately calibrated hydraulic models and exact measurements of demand

#### 3.2.2 Advanced signal processing and feature extraction

Raw sensor data, especially high-frequency acoustic and vibration signals, need to be transformed to extract discriminative features that a machine learning model can effectively utilize (Martinez-Rios et al., 2024; Saravanabalaji et al., 2023). The different ways of performing this can be categorized as time frequency transformation, feature engineering, and dimensionality reduction.

##### 3.2.2.1 Time-frequency transformation

Due to leak signals often being non-stationary, methods that utilize and analyze data in both time and frequency domains can be beneficial. Examples of this can be seen in the Fast Fourier Transform (FFT), a technique used in literature (Saravanabalaji et al., 2023) that uses and computes Discrete Fourier Transform (DFT) to convert the collected acoustic signals from their original time domain to the frequency domain, whereas (Liu et al., 2024), carried out a Short Time Fourier Transform (STFT) to create spectrograms of collected acoustic signals and analyze the difference between signals from leaking pipes and those from normal, non-leaking pipes. An advanced method like the Continuous Wavelet Transform (CWT) can be used to generate 2D scalograms from 1D acoustic signals that are treated as images for analysis by CNN models carried out by (Atanane et al. 2023). A particularly powerful technique is the wavelet scattering transform (WST), which creates a stable signal representation and is invariant with translation, enabling the extraction of robust features without needing manual feature engineering or even complex deep learning models (Martinez-Rios et al., 2024).

##### 3.2.2.2 Feature engineering and dimensionality reduction

Once signals are transformed, the relevant features must be selected to build an effective model. One approach involves engineering a large set of features from the time series and power spectral density data and then ranking them to select the most important ones (Ravichandran et al., 2021). To manage the high dimensionality that can result from these transformations, techniques like Principal Component Analysis (PCA) are employed to reduce the number of features while retaining the most important information (Quinõnes-Grueiro et al., 2018; Saravanabalaji et al., 2023). An alternative, more integrated approach is to use deep learning models like the domain-informed VAE, which can help to reduce the dimensionality of the input data (e.g., a 24-h flow profile) into a low-dimensional latent space that efficiently captures the distinct characteristics of leakage and non-leakage events (McMillan et al., 2024).

### 3.3 Performance and effectiveness

The performance of the advanced models in literature is great, demonstrating high accuracy in both detecting the presence of a leak and pinpointing its location, as well as having a good attempt at identifying small or early-stage leaks that conventional methods often miss.

#### 3.3.1 High accuracy for leak detection

For the task of binary classification, between distinguishing leak and non-leak conditions, both MEL and hybrid deep learning models have achieved exceptional results. For example, an ensemble method that combined six different techniques achieved a maximum detection accuracy of 99.3% in a laboratory setting (Saravanabalaji et al., 2023), whereas in the same domain (Ravichandran et al., 2021) reduced false positive rates to 0.16% whilst achieving an overall accuracy of 99.84% using a mixed dataset of real-world and augmented simulations. With hybrid deep learning methods, a framework using a domain-informed variational autoencoder (VAE) combined with a Support Vector Machine (SVM) demonstrated 98% classification accuracy across a real-world dataset of over 2,500 District Metered Areas (DMAs) (McMillan et al., 2024). Another deep learning model had also shown strong performance, with an EfficientNet Convolutional Neural Network (CNN) attaining 97.45% testing accuracy on an acoustic dataset (Atanane et al., 2023) with the use of cost-effective TinyML techniques.

The ability to detect small leaks in WDN is a critical benchmark for advanced models. Neural networks have proven particularly effective, successfully detecting intra-domestic leaks as small as ≤ 1 L/h with an accuracy between 92% and 96% (Zese et al., 2021). Even smaller leaks have been identified in various settings, which can be shown in the study carried out by (Qi et al. 2024), where a set of specialized hardware, such as in-pipe robots, has also shown promise, using pressure difference sensors to detect a leak of 0.083 L/s on a PVC pipe.

#### 3.3.2 High efficacy leak location

In addition to detecting leaks, the efficacy of a model is measured by its ability to not only identify the problem but also to locate the issue to solve, which is where the location of leaks shows significance. An example of this is highlighted through the study (Rajabi et al., 2023), which achieved an accuracy of approximately 70% in a real-time environment, although it does not demonstrate high performance accuracy, it shows real word attempt in solving the locating of leaks for possible repairs. (Lucin et al. 2021) demonstrated that a Random Forest classifier, even when trained only on leaks occurring at network nodes, could narrow down the location of leaks occurring along pipe segments by identifying the top 3 to 5 most suspect nodes. This highlights the ability of machine learning to generalize from simulated data to more realistic scenarios.

Other hybrid approaches have also yielded strong localization results. A method combining deep learning with clustering was found to qualitatively and quantitatively outperform a traditional model-based localization approach (Romero et al., 2022, *cited* in Romero-Ben et al., 2023). Similarly, a leak localization scheme using a Bayesian classifier outperformed both k-Nearest Neighbors (k-NN) and angle-based methods, especially when temporal reasoning was applied over a longer time horizon to reduce the impact of noise and uncertainty (Soldevila et al., 2017). These studies show that while detection focuses on a binary outcome, localization benefits from classifiers that can effectively differentiate between unique pressure or acoustic signatures produced by leaks at different points in the network.

Table 7 provides a comparative synthesis of best best-performing models identified in the review.

**Table 7:** Comparative synthesis among best best-performing models.

References	Model type	Data modality used	Dataset type	Feature extraction technique	Performance metric (%)
(Leonzio et al. 2025)	Hybrid deep learning (Sensor fusion)	Acoustic & vibration	Real world & laboratory testbed	Time frequency transformation using STFT technique + complex-valued CNN	99
(Ma 2024)	Hybrid deep learning (T-LSTM)	Pressure	Real world	Feature engineering using sequential learning through neighboring data points	98.3
(McMillan et al. 2024)	Hybrid deep learning (VAE-SVM)	Flow	Real world	Dimensionality reduction using domain-informed VAE	98.2
(Ravichandran et al. 2021)	Ensemble (GBT)	Acoustic	Simulated	Dimensionality reduction using PCA	99.84
(Saravanabalaji et al. 2023)	Ensemble (combination of LR, XGBoost, SVM, RF, AdaBoost, and NN)	Acoustic	Laboratory testbed	Time frequency transformation using FFT and dimensionality reduction using PCA	99.3

### 3.4 Use of XAI in hybrid water leak detection

Although high accuracy performance and resilience of a model's capability are essential for its implementation in a real-world environment, the “black box” nature of a model can be a significant barrier to adoption in critical infrastructure management, as operators must be able to trust and understand the predictions of the system to act effectively. Not much literature has covered this aspect. The limitations of methodologies that do not incorporate XAI are significant. Without transparency, it can be difficult for operators to understand the underlying reasons for the predictions of a model, compromising trust and leading to hesitation in responding to automated alarms (Chan et al., 2018).

In the high-stakes environment of a Water Distribution Network (WDN), where a false alarm leads to wasted resources and a missed detection results in continued water loss, transparency is crucial for building operator trust and bridging the gap between complex AI and human decision-makers (Naith and Mancy, 2025). To address this, a growing interest in integrating XAI, with SHapley Additive exPlanations (SHAP) emerges as a prominent and effective methodology, which is shown in the researchers' study. For example, a hybrid framework combining Multi-Agent Deep Reinforcement Learning (MADRL) with SHAP provides human-understandable explanations for why a DRL agent made a specific control decision, such as adjusting a valve, by attributing the action to specific input signals like pressure or flow changes (Naith and Mancy, 2025). Their framework demonstrated high reliability, achieving an average SHAP consistency score of 88% across various scenarios, including leak events. (Naith and Mancy 2025) further elaborated that providing clear justifications for a model's predictions, an XAI-integrated system significantly enhances overall efficacy, like when an alarm is triggered, where SHAP can highlight the specific sensor readings that influenced the decision, allowing an operator to verify the model's reasoning against their own expertise. This transforms the model from an opaque tool into a trustworthy decision-support system, empowering operators with actionable insights and enabling more confident, rapid responses that improve the efficiency of the entire leak management workflow.

### 3.5 Knowledge gaps and controversies

**Figure 5:**
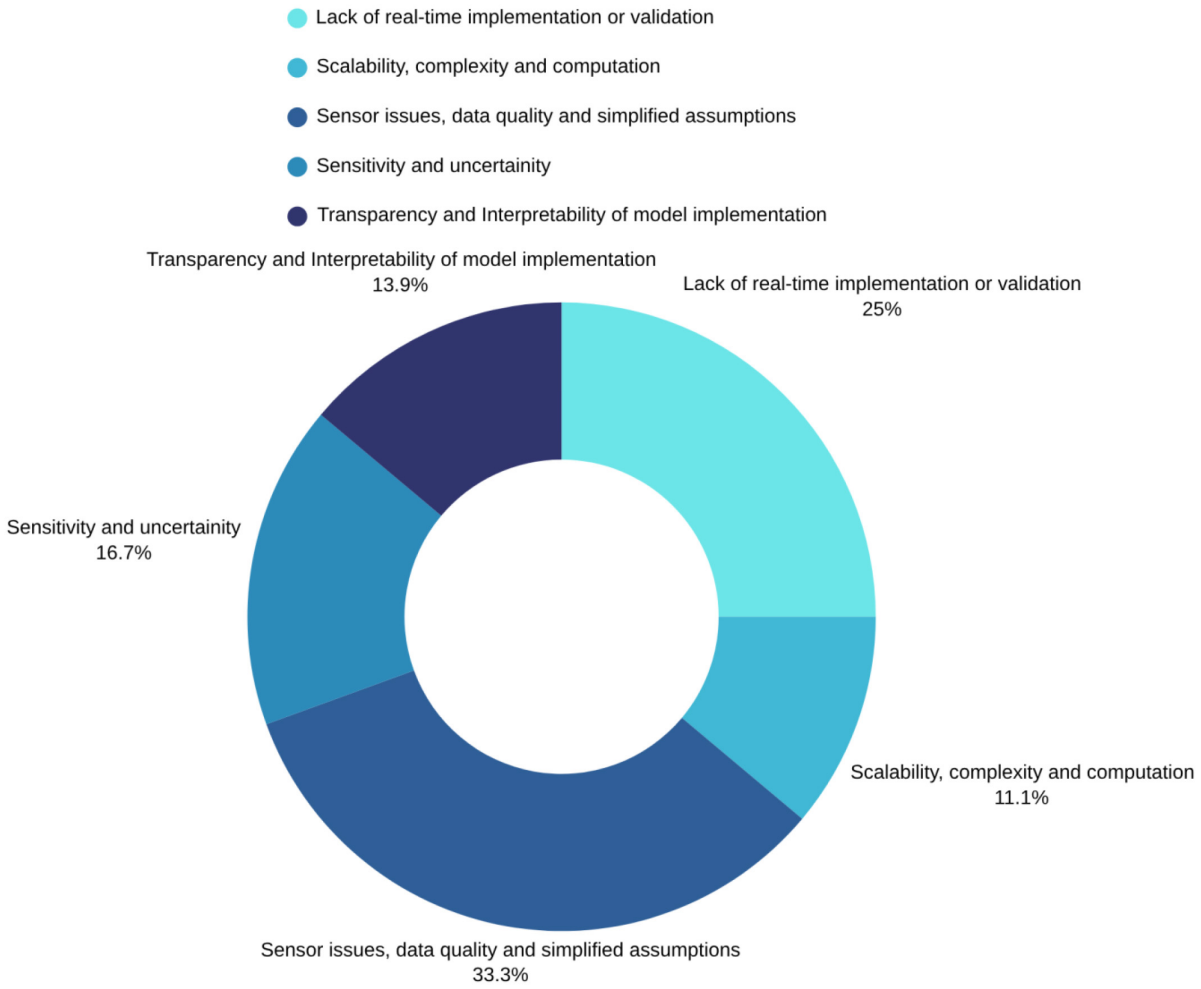
Summary of knowledge gaps identified in the literature.

Despite witnessing the essential need for transparent interpretability in water leak detection models, several persistent knowledge gaps were also identified. Figure 5 visualizes the gaps identified in the water leak detection and management body of knowledge.

° **Sensor Issues, Data Quality, and Simplified Assumptions** were the most prevalent challenges. Many studies rely on idealized or simulated data, which do not reflect real-world conditions (Chan et al., 2018; Naith and Mancy, 2025). For example, some models make unrealistic assumptions about network homogeneity or the absence of initial leaks (Chan et al., 2018; Rajabi et al., 2023; Romero-Ben et al., 2023; Soldevila et al., 2017). The performance of methods that use acoustic or hydrophone data is often negatively impacted by background noise, an issue frequently encountered in operational environments (Chan et al., 2018; Martinez-Rios et al., 2024). Other studies note that the use of laboratory-scale testbeds or simulated data limits the direct applicability of their findings to complex, real-world WDNs (Atanane et al., 2023; Rajan and Li, 2025 Saravanabalaji et al., 2023). Furthermore, data scarcity (Menapace et al., 2020) and the high cost of deploying a sufficient number of sensors remains a major constraint for data-driven approaches (Moubayed et al., 2021; Romero-Ben et al., 2023), which may be the reasons for poor data quality and simplified assumptions witnessed in several studies.
° **Sensitivity and Uncertainty** show that a significant number of methodologies struggle with leak sensitivity, particularly in detecting small or incipient leaks (Chan et al., 2018; Zese et al., 2021). Moreover, many models fail to adequately handle the uncertainty inherent in WDNs, such as the stochastic nature of water demand (Lucin et al., 2021), which can lead to high false alarm rates (Tornyeviadzi et al., 2023). This issue is compounded when models trained on simulated data show reduced accuracy when faced with real-world demand variations and sensor measurement ambiguity (McMillan et al., 2024; Muniz Do Nascimento and Gomes-Jr., 2023).
° **Lack of Real-Time Implementation or Validation:** A critical gap exists between theoretical model development and practical real-time application (Leonzio et al., 2025; Muniz Do Nascimento and Gomes-Jr., 2023; Rajan and Li, 2025; Xu et al., 2025). Many proposed frameworks are validated using benchmark datasets or in offline simulations, with researchers explicitly stating the need for future deployment in live operational settings to test performance against real-world challenges like sensor faults and data delays (Martinez-Rios et al., 2024; Naith and Mancy, 2025; Saravanabalaji et al., 2023). This gap highlights a crucial missing step in translating academic research into deployable solutions for water utilities.
**Scalability, Complexity, and Computation:** The complexity of advanced models presents scalability challenges. For instance, some optimization methods may not scale well to large WDNs (Moubayed et al., 2021; Romero-Ben et al., 2023). Combining more models can increase overall model complexity and computational cost, especially in high-dimensional or large-scale networks. For instance, computing SHAP values in multi-agent settings can be expensive and pose scalability challenges for networks with over 1000 nodes (Naith and Mancy, 2025), additionally computationally expensive components, such as the SHAP algorithm for explainability in complex multi-agent systems, can hinder real-time performance and scalability. Whereas, a disadvantage of late fusion strategies is that considering a separate model for each data modality increases overall model complexity (Martinez-Rios et al., 2024).

- **Performance and interpretability**: While using multiple base learners in ensemble methods can improve performance and reduce error rates (Zhou et al., 2021), studies have demonstrated that there isn't always a constant proportional increase in accuracy with more generated samples (Liu et al., 2024), and a single well refined model can sometimes outperform a general ensemble model (Ma, 2024).
- **Balancing components for large WDNs**: For large water networks, hybrid algorithm performance may deteriorate due to imbalance in component algorithms, highlighting that composition and balance are critical over a strict number (Wang et al., 2014). The optimal number of combined algorithms, therefore, depends on balancing improved performance against increased complexity, computational demands and the need for interpretability.

° **Transparency and interpretability of Models:** As identified earlier, a significant knowledge gap in water leak detection revolves around the transparency and interpretability of advanced machine learning models (Naith and Mancy, 2025). While techniques like Artificial Neural Networks (ANNs) and other deep learning architectures demonstrate high accuracy, their “black-box” nature makes it difficult to understand the underlying reasons for their predictions, which is crucial for gaining user trust and acceptance in practical applications (McMillan et al., 2024; Moubayed et al., 2021). This lack of interpretability creates a dilemma in which operators must often choose between suboptimal but understandable strategies and complex, accurate but opaque solutions. To address this, recent research has focused on integrating Explainable AI (XAI) to bridge the gap between complex models and human operators, which was demonstrated by (Naith and Mancy 2025). Similarly, other studies are beginning to incorporate visualization techniques like the MGrad-CAM to interpret the decision-making process of CNN models, highlighting which features in the input data were most critical for a prediction (Leonzio et al., 2025). Filling this research gap is essential to make intelligent leak detection systems truly trustworthy and actionable for water utility operators.

### 3.6 A transparent hybrid ML conceptual framework for water leak detection

After identifying gaps in the research field of hybrid ML models for water leak detection, a generalized conceptual framework can be drawn.

**Figure 6:**
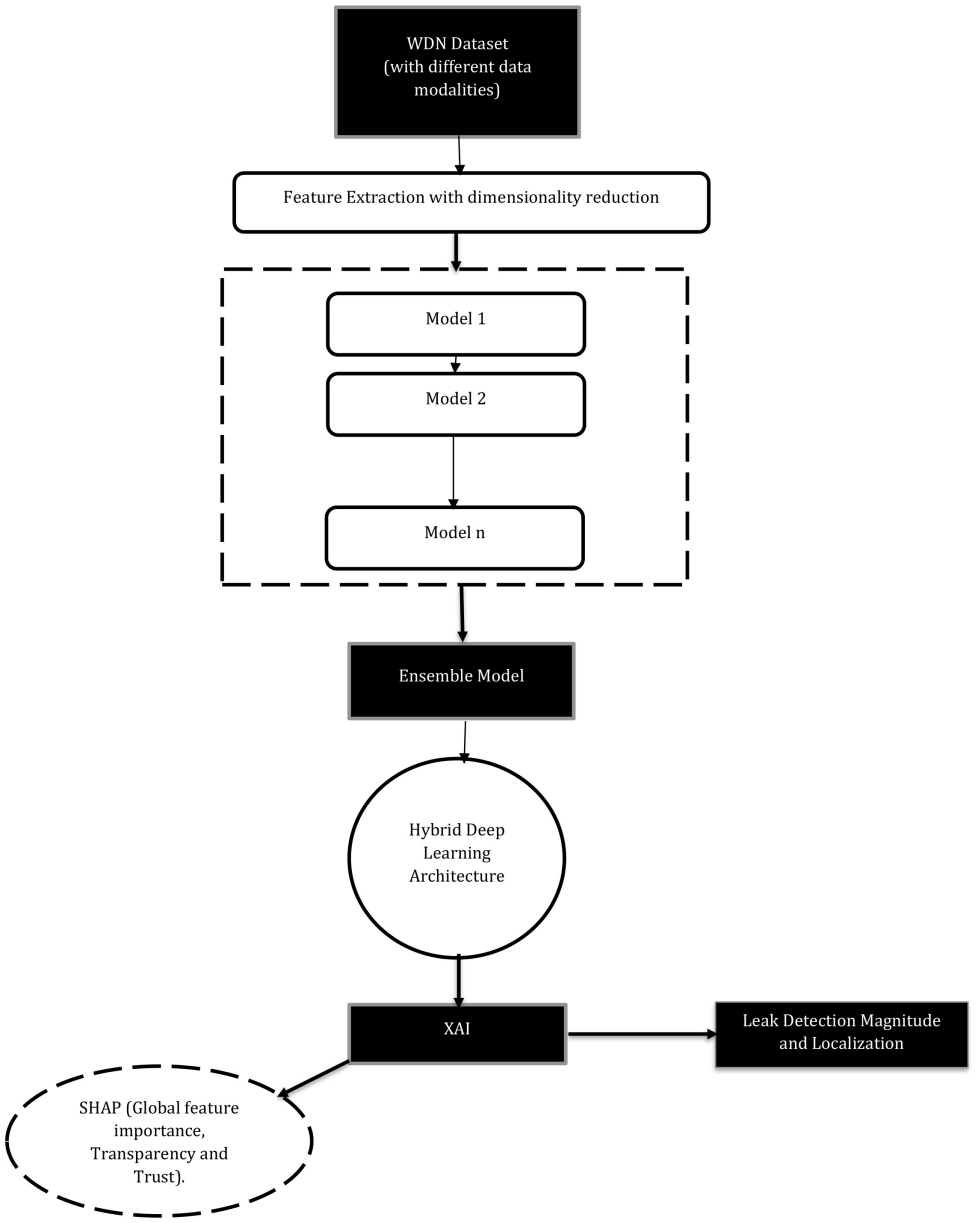
A transparent hybrid ML conceptual framework for leak detection.

With the proposed conceptual framework in Figure 6, it integrates different data modalities, feature extraction, and dimensionality reduction for computational cost, a deep hybrid ensemble learning architecture, and XAI to deliver a robust and transparent system for water leak detection, localization, and magnitude assessment.

#### 3.6.1 WDN dataset

A fundamental principle of the framework is that a single data modality is often insufficient to capture the complex nature of leakage under varying operational conditions (Martinez-Rios et al., 2024; Moubayed et al., 2021). By combining complementary information, the model will improve its detection and classification accuracy (Leonzio et al., 2025).

#### 3.6.2 Feature extraction with dimensionality reduction

To manage the high dimensionality and complexity of time-series data, the framework employs a feature extraction technique that incorporates dimensionality reduction because a direct application of classifiers to high-dimensional raw data can be computationally inefficient and can lead to underfitting, as the model may struggle to distinguish critical leak patterns from noise. Therefore, dimensionality reduction is a pivotal strategy in the conceptual framework as it may incorporate the high computational cost of SHAP values, which may lead to scalability, complexity, and computational issues (Moubayed et al., 2021; Naith and Mancy, 2025).

#### 3.6.3 A deep hybrid and MEL architecture

The core of the framework is a deep hybrid and MEL architecture, which combines multiple models to improve accuracy, robustness, and generalization. As seen in the literature, the models that yielded the best results included both hybrid deep learning and MEL methods, tabulated in Table 7.

#### 3.6.4 Integration of XAI

A critical challenge with complex models and deep learning architectures is their “black box” nature, which can hinder adoption and trust among operators. The framework directly addresses this gap by integrating an XAI component based on Shapley Additive Explanations (SHAP), as shown in the study carried out by (Naith and Mancy 2025). It will incorporate this by **Feature Attribution and Transparency**, where SHAP is applied as a *post-hoc* analysis method to interpret the model's decisions and attribute the action outputs to the specific input features (e.g., pressure, acoustic signals, etc.) that influenced them, providing human-understandable explanations. This hopes to bridge the gap between complex AI models and human operators by offering transparent insights into the model's functionality. It will also incorporate this by **Global Importance and Trustworthiness**, where the framework uses SHAP to analyze global feature importance, allowing operators to understand which factors matter most to the model overall.

#### 3.6.5 Multi-faceted output: detection, magnitude, and localization

The final output of the framework is not a single decision but a multi-faceted assessment that hopes to provide comprehensive information for leak management. With the **Leak Detection** output, the primary output is a binary classification decision indicating the presence or absence of a leak. With leak magnitude classification, the conceptual framework hopes to identify small or early leaks in a WDN, keeping in mind the type of data modality that could help with this functionality, as discussed earlier. The framework also hopes to provide a probable location for the leak once a leak has been identified.

## 4 Critical discussion

This systematic review synthesizes findings from 27 studies to address the primary research question: *How can a transparent hybrid machine learning framework enhance the accuracy and efficacy of detecting and localizing leaks and early leaks in a Water Distributed System as compared to single machine learning models and conventional methods?*

With water leak management framed within the context of promoting sustainability, resilience, and ensuring future water security aligns broadly with the goal of SDG 6, reducing leakage is essential for ensuring sustainable and resilient water supply systems (McMillan et al., 2024) due to leaks exacerbating water scarcity challenges and posing risks of contamination, which affects public health and safe water access (Atanane et al., 2023; Romero-Ben et al., 2023). Intelligent data-driven methods and smart water management (SWM) infrastructure, with its metering system, are presented as crucial for optimizing WDNs and improving sustainability metrics (Rajan and Li, 2025) where the management of NRW is recognized as vital for the sustainability and efficient functioning of urban water systems (Romero-Ben et al., 2023) which water utilities and municipalities can adopt.

In the review articles, (Chan et al. 2018) provided a broad overview, categorizing existing leak detection technologies into passive (e.g., visual, acoustic) and active systems (which include model-based and data-driven approaches), whereas (Romero-Ben et al., 2023) narrowed the focus to software-based methods operating under steady-state conditions, directly comparing model-based vs. data-driven approaches for localization. (Moubayed et al. 2021) surveyed hardware in the loop-based leak detection methods, simulation in the loop-based techniques, and hybrid methods, but its main contribution was proposing sensor data fusion and federated learning paradigms to improve efficiency and privacy, as it is especially needed in industrial leak detection. (Qi et al. 2024) touched on the detection of small leaks that compared techniques utilized in pipe robots vs. continuous sensor networks, whereas (Rajan and Li, 2025) offered a specific review that focuses exclusively on flow data-based techniques and emphasized the necessity of integrating these into fully automated leak management systems beyond just detection.

The analysis reveals a trend toward hybrid deep learning and ensemble methods, which demonstrably enhance detection accuracy by combining the complementary strengths of multiple algorithms. These approaches consistently outperform single framework models and seemingly mixed model-based or data-driven models by leveraging diverse data modalities and mitigating the weaknesses of individual classifiers (Leonzio et al., 2025; Martinez-Rios et al., 2024; Romero-Ben et al., 2023). The performance metrics reported in the literature are a testament to this, with various hybrid models achieving exceptional classification accuracies of 98.2%, 99.3%, and even 99.84% in distinguishing leak and non-leak events (Leonzio et al., 2025; Ma, 2024; McMillan et al., 2024; Ravichandran et al., 2021; Saravanabalaji et al., 2023). This shift away from traditional model-based methods toward more robust data-driven and hybrid systems is a logical response to the inherent complexity and uncertainty of real-world WDNs, which purely hydraulic models often struggle to capture (Chan et al., 2018).

However, a central argument of this review is that high accuracy alone is insufficient to guarantee practical efficacy. The sophisticated nature of deep learning and ensemble models often results in “black box” systems whose decision-making processes are opaque to human operators. This lack of transparency undermines operator trust, creating a significant barrier to the adoption of these technologies in high-stakes environments or critical water management infrastructures (Naith and Mancy, 2025). When an operator cannot understand *why* a model has flagged an anomaly, they are less likely to act on the alert, thereby invalidating the model's high accuracy.

This finding marks a critical point where transparency becomes vital. The integration of XAI, using techniques such as SHAP, directly addresses this challenge by making model predictions interpretable (Naith and Mancy, 2025). By providing clear feature-based justifications for its outputs, an XAI-enabled hybrid framework transforms from an unclear tool into a reliable and trustworthy decision-support system. This transparency empowers operators to act on alerts with confidence and speed, which will improve the entire leak management workflow from detection to repair. Therefore, a transparent hybrid framework enhances not only accuracy through algorithmic implementations but, more importantly, elevates real-world efficacy by including human-AI collaboration, which is necessary for effective and operational decision-making.

## 5 Future directions

Building on the knowledge gaps identified throughout this review, several key areas for future research emerge as critical to advance the development of practical and deployable hybrid leak detection systems, especially in complex and unpredictable environments.

First, the most pressing need is to bridge the gap **from simulation to reality**. A significant portion of the reviewed literature validates models using simulated or laboratory-based data, and there is a critical lack of real-world implementation and validation (Rajan and Li, 2025). Future research must prioritize the deployment of these models in live WDNs to test their robustness against noisy data, possible sensor failures, and unpredictable operational dynamics. This includes exploring resource-efficient solutions (Moubayed et al., 2021), for example, solutions such as TinyML, which enable real-time, on-device processing and can facilitate wider adoption in resource-constrained utilities (Atanane et al., 2023).

Second, future work must continue to **address data scarcity and quality**. Given that high-quality labeled leak data is rare (Menapace et al., 2020). Research should focus more on advancing data augmentation techniques. Methods like Generative Adversarial Networks (GANs) show promise in creating diverse and realistic synthetic training datasets that can improve model performance and robustness (Liu et al., 2024; Rajabi et al., 2023). Simultaneously, developing models that are inherently resilient to the imbalanced and noisy data characteristic of real-world WDNs is essential for reducing false alarms and improving reliability (Tornyeviadzi et al., 2023).

Third, as models become more complex and their application scales to larger networks, **enhancing scalability and computational efficiency** is crucial. The computational cost of some advanced models and their associated explainability algorithms can be a barrier to real-time application (Moubayed et al., 2021; Naith and Mancy, 2025; Romero-Ben et al., 2023). Future research should focus on optimizing algorithms for large-scale WDNs and developing more computationally efficient methods to achieve framework transparency without compromising real-time performance in future models.

Finally, XAI integration represents a vital but emerging field that requires deepening transparency and trust. Future research must move beyond simply acknowledging the need for explainability. It should actively explore and compare different XAI techniques, establish standardized metrics to measure interpretability, and, most importantly, conduct human-in-the-loop studies. Such studies are necessary to validate whether the explanations provided by XAI truly build operator trust, improve decision-making, and ultimately enhance the overall efficacy of the leak management system (Naith and Mancy, 2025).

## 6 Conclusion

This systematic review has addressed the critical global challenge of water loss by evaluating the landscape of advanced hybrid machine learning models for leak detection in WDNs. The core findings show that recent advances in water leakage detection increasingly rely on hybrid and ensemble learning methods. While such approaches are reported across the literature, their real-world effectiveness remains closely tied to operator trust and system interpretability. The inherent complexity and “black-box” nature of the models continues to pose challenges for practical deployment, highlighting the need for explainable mechanisms that bridge high predictive capabilities with operational usability.

The primary contribution and uniqueness of this review lie in its dual focus on both predictive performance and the essential, yet frequently overlooked, requirement for transparency. By framing Explainable AI not as a secondary feature but as a core component for operational success, this review addresses a primary barrier to the real-world adoption of AI in critical infrastructure management. The analysis concludes that a transparent hybrid framework will enhance leak detection accuracy through the synergy of its combined algorithms, while simultaneously elevating its practical efficacy by integrating XAI. This integration provides the interpretability required for human operators to trust, understand, and decisively act upon the model's outputs.

Ultimately, the future of intelligent water management depends on our ability to create systems that are not only powerful but also collaborative. The next generation of these systems must be designed as human-centric, trustworthy, and transparent tools. Only by achieving this between machine intelligence and human expertise can we realize the full potential of AI in securing a sustainable and water-resilient future.

## Data Availability

The original contributions presented in the study are included in the article/supplementary material, further inquiries can be directed to the corresponding author.

## References

[B1] Aghashahi M. Sela L. Banks M. K. (2023). Benchmarking dataset for leak detection and localization in water distribution systems. Data Brief 48:109148. doi: 10.17632/tbrnp6vrnj.1 37128586 PMC10147960

[B2] Alcamo J. Henrichs T. Rösch T. (2000). World Water in 2025-Global Modeling and Scenario Analysis for the World Commission on Water for the 21st Century. Kassel World Water Series.

[B3] Atanane O. Mourhir A. Benamar N. Zennaro M. (2023). Smart buildings: water leakage detection using TinyML. Sensors 23:9210. doi: 10.3390/s23229210 38005596 PMC10675406

[B4] Azevedo B. F. Rocha A. M. A. C. Pereira A. I. (2024). Hybrid approaches to optimization and machine learning methods: a systematic literature review. Mach. Learn. 113, 4055–4097. doi: 10.1007/s10994-023-06467-x

[B5] Bakhtiari V. Kerchi H. D. Piadeh F. Behzadian K. Nasirzadeh F. (2025). Role of the internet of things in flood risk management: a critical review on current practices and future directions. Natural Hazards 121, 19473–19505. doi: 10.1007/s11069-025-07589-2

[B6] CASP Checklists (2025). Critical Appraisal Skills Programme [WWW Document]. Available online at: https://casp-uk.net/casp-tools-checklists/ (Accessed October 10, 2025).

[B7] Chan T. K. Chin C. S. Zhong X. (2018). Review of current technologies and proposed intelligent methodologies for water distributed network leakage detection. IEEE Access 6, 78846–78867. doi: 10.1109/ACCESS.2018.2885444

[B8] Dogani A. Dourandish A. Ghorbani M. Shahbazbegian M. R. (2020). A hybrid meta-heuristic for a bi-objective stochastic optimization of urban water supply system. IEEE Access 8, 135829–135843. doi: 10.1109/ACCESS.2020.3009885

[B9] El-Zahab S. Zayed T. (2019). Leak detection in water distribution networks: an introductory overview. Smart Water 4:5. doi: 10.1186/s40713-019-0017-x

[B10] Goldberg D. E. (1989). Genetic Algorithms in Search, Optimization, and Machine Learning. Reading, MA: Addison-Wesley Pub. Co. Available online at: https://www2.fiit.stuba.sk/~kvasnicka/Free%20books/Goldberg_Genetic_Algorithms_in_Search.pdf

[B11] Islam M. R. Azam S. Shanmugam B. Mathur D. (2022). A review on current technologies and future direction of water leakage detection in water distribution network. IEEE Access 10, 107177–107201. doi: 10.1109/ACCESS.2022.3212769

[B12] Kumari U. Swamy K. Gupta A. Karri R. R. Meikap B. C. (2021). “Global water challenge and future perspective,” in Green Technologies for the Defluoridation of Water, eds. M. H. Dehghani, R. Karri, and E. Lima (Amsterdam: Elsevier). doi: 10.1016/B978-0-323-85768-0.00002-6

[B13] Leonzio D. U. Mandelli S. Bestagini P. Marcon M. Tubaro S. (2025). Water leak detection and classification with complex-valued neural networks and sensor fusion. IEEE Access 13, 115669–115685. doi: 10.1109/ACCESS.2025.3585306

[B14] Liemberger R. Wyatt A. (2019). Quantifying the global non-revenue water problem. Water Supply 19, 831–837. doi: 10.2166/ws.2018.129

[B15] Liu R. Zayed T. Xiao R. (2024). Advanced acoustic leak detection in water distribution networks using integrated generative model. Water Res. 254:121434. doi: 10.1016/j.watres.2024.121434 38484549

[B16] Lucin I. Carija Z. Druzeta S. Lucin B. (2021). Detailed leak localization in water distribution networks using random forest classifier and pipe segmentation. IEEE Access 9, 155113–155122. doi: 10.1109/ACCESS.2021.3129703

[B17] Ma T. (2024). “Data-driven leak detection and identification in water distribution networks using transductive long short-term memory,” in 2nd IEEE International Conference on Data Science and Information System, ICDSIS 2024 (Institute of Electrical and Electronics Engineers Inc). doi: 10.1109/ICDSIS61070.2024.10594295

[B18] Martinez-Rios E. A. Barrientos D. Bustamante R. (2024). Water leakage classification with acceleration, pressure, and acoustic data: leveraging the wavelet scattering transform, unimodal classifiers, and late fusion. IEEE Access 12, 84923–84951. doi: 10.1109/ACCESS.2024.3416056

[B19] McMillan L. Fayaz J. Varga L. (2024). Domain-informed variational neural networks and support vector machines based leakage detection framework to augment self-healing in water distribution networks. Water Res. 249:120983. doi: 10.1016/j.watres.2023.120983 38118223

[B20] Menapace A. Zanfei A. Felicetti M. Avesani D. Righetti M. Gargano R. . (2020). Burst detection in water distribution systems: the issue of dataset collection. Appl. Sci. 10, 1–19. doi: 10.3390/app10228219

[B21] Moubayed A. Sharif M. Luccini M. Primak S. Shami A. (2021). Water leak detection survey: challenges research opportunities using data fusion federated learning. IEEE Access 9, 40595–40611. doi: 10.1109/ACCESS.2021.3064445

[B22] Muniz Do Nascimento W. Gomes-Jr L. (2023). Enabling low-cost automatic water leakage detection: a semi-supervised, autoML-based approach. Urban Water J. 20, 1471–1481. doi: 10.1080/1573062X.2022.2056710

[B23] Naith Q. H. Mancy H. (2025). An IoT-enabled hybrid DRL-XAI framework for transparent urban water management. Comput. Model. Eng. Sci. 144, 387–405. doi: 10.32604/cmes.2025.066917

[B24] PRISMA (2020). PRISMA Statement. Available online at: https://www.prisma-statement.org/

[B25] Puust R. Kapelan Z. Savic D. A. Koppel T. (2010). A review of methods for leakage management in pipe networks. Urban Water J. 7, 25–45. doi: 10.1080/15730621003610878

[B26] Qi R. Cao M. Yntema D. R. (2024). Recent developments of subsurface small-leak detection techniques in water distribution networks: a review. IEEE Robot. Autom. Mag. 31, 108–118. doi: 10.1109/MRA.2024.3351483

[B27] Quinõnes-Grueiro M. Verde C. Prieto-Moreno A. Llanes-Santiago O. (2018). An unsupervised approach to leak detection and location in water distribution networks. Int. J. Appl. Math. Comput. Sci. 28, 283–295. doi: 10.2478/amcs-2018-0020

[B28] Rajabi M. M. Komeilian P. Wan X. Farmani R. (2023). Leak detection and localization in water distribution networks using conditional deep convolutional generative adversarial networks. Water Res. 238:120012. doi: 10.1016/j.watres.2023.120012 37150062

[B29] Rajan G. Li S. (2025). A systematic literature review on flow data-based techniques for automated leak management in water distribution systems. Smart Cities 8:78. doi: 10.3390/smartcities8030078

[B30] Ravichandran T. Gavahi K. Ponnambalam K. Burtea V. Mousavi J. S. (2021). Ensemble-based machine learning approach for improved leak detection in water mains. J. Hydroinf. 23, 307–323. doi: 10.2166/hydro.2021.093

[B31] Romano M. Kapelan Z. Savić D. A. (2014). Automated detection of pipe bursts and other events in water distribution systems. J. Water Resour. Plan Manage. 140, 457–467. doi: 10.1061/(ASCE)WR.1943-5452.0000339

[B32] Romero L. Blesa J. Puig V. Cembrano G. (2022). Clustering-learning approach to the localization of leaks in water distribution networks. J. Water Resourc. Plann. Manage. 148:04022003. doi: 10.1061/(ASCE)WR.1943-5452.0001527

[B33] Romero-Ben L. Alves D. Blesa J. Cembrano G. Puig V. Duviella E. . (2023). Leak detection and localization in water distribution networks: review and perspective. Annu. Rev. Control 55, 392–419. doi: 10.1016/j.arcontrol.2023.03.012

[B34] Saeidmehr A. Steel P. D. G. Samavati F. F. (2024). Systematic review using a spiral approach with machine learning. Syst. Rev. 13:32. doi: 10.1186/s13643-023-02421-z 38233959 PMC10792832

[B35] Saravanabalaji M. Sivakumaran N. Ranganthan S. Athappan V. (2023). Acoustic signal-based water leakage detection system using hybrid machine learning model. Urban Water J. 20, 1123–1139. doi: 10.1080/1573062X.2023.2239782

[B36] Soldevila A. Fernandez-Canti R. M. Blesa J. Tornil-Sin S. Puig V. (2017). Leak localization in water distribution networks using Bayesian classifiers. J. Process Control 55, 1–9. doi: 10.1016/j.jprocont.2017.03.015

[B37] Tornyeviadzi H. M. Mohammed H. Seidu R. (2023). Robust night flow analysis in water distribution networks: a BiLSTM deep autoencoder approach. Adv. Eng. Inf. 58:102135. doi: 10.1016/j.aei.2023.102135

[B38] Ulusoy A. J. Mahmoud H. A. Pecci F. Keedwell E. C. Stoianov I. (2022). Bi-objective design-for-control for improving the pressure management and resilience of water distribution networks. Water Res. 222:118914. doi: 10.1016/j.watres.2022.118914 35933815

[B39] Vörösmarty C. J. Green P. Salisbury J. Lammers R. B. (2000). Global water resources: vulnerability from climate change and population growth. Science 289, 284–288. doi: 10.1126/science.289.5477.284 10894773

[B40] Wang Q. Savic D. A. Kapelan Z. (2014). Hybrid metaheuristics for multi-objective design of water distribution systems. J. Hydroinf. 16, 165–177. doi: 10.2166/hydro.2013.009

[B41] Wolpert D. H. Macready W. G. (1996). No Free Lunch Theorems for Search. Available online at: https://www.researchgate.net/publication/221997149_No_Free_Lunch_Theorems_for_Search

[B42] Wordsworth C. (2023). How Digital Analytics Can Help Locate and Detect Leaks. WaterWorld. Home [online]. Available online at: https://www.waterworld.com/ (accessed October 2025).

[B43] Xing L. Sela L. (2019). Unsteady pressure patterns discovery from high-frequency sensing in water distribution systems. Water Res. 158, 291–300. doi: 10.1016/j.watres.2019.03.051 31051374

[B44] Xu J. Yuan X. Zheng L. Lin D. (2025). Hyperclustering: high-order deep/shallow feature clustering for subway shield tunneling water leakage detection. IEEE Access 13, 88328–88341. doi: 10.1109/ACCESS.2025.3571523

[B45] Zese R. Bellodi E. Luciani C. Alvisi S. (2021). Neural network techniques for detecting intra-domestic water leaks of different magnitude. IEEE Access 9, 126135–126147. doi: 10.1109/ACCESS.2021.3111113

[B46] Zhou M. Yang Y. Xu Y. Hu Y. Cai Y. Lin J. . (2021). A pipeline leak detection and localization approach based on ensemble TL1DCNN. IEEE Access 9, 47565–47578. doi: 10.1109/ACCESS.2021.3068292

